# Analysis of Three Compounds in Flos Farfarae by Capillary Electrophoresis with Large-Volume Sample Stacking

**DOI:** 10.1155/2017/3813879

**Published:** 2017-09-05

**Authors:** Hai-xia Yu, Zeng-Yan Hao, Lu Li, Ya-yun Huang, Hong-Fen Zhang, An-jia Chen

**Affiliations:** ^1^Translational Medicine Research Center, Shanxi Medical University, Taiyuan 030001, China; ^2^Pharmacy Department, Shanxi Boai Hospital, Taiyuan 030001, China; ^3^School of Pharmacy, Shanxi Medical University, Taiyuan 030001, China

## Abstract

The aim of this study was to develop a method combining an online concentration and high-efficiency capillary electrophoresis separation to analyze and detect three compounds (rutin, hyperoside, and chlorogenic acid) in Flos Farfarae. In order to get good resolution and enrichment, several parameters such as the choice of running buffer, pH and concentration of the running buffer, organic modifier, temperature, and separation voltage were all investigated. The optimized conditions were obtained as follows: the buffer of 40 mM NaH_2_P0_4_-40 mM Borax-30% v/v methanol (pH 9.0); the sample hydrodynamic injection of up to 4 s at 0.5 psi; 20 kV applied voltage. The diode-array detector was used, and the detection wavelength was 364 nm. Based on peak area, higher levels of selective and sensitive improvements in analysis were observed and about 14-, 26-, and 5-fold enrichment of rutin, hyperoside, and chlorogenic acid were achieved, respectively. This method was successfully applied to determine the three compounds in Flos Farfarae. The linear curve of peak response versus concentration was from 20 to 400 *µ*g/ml, 16.5 to 330 *µ*g/mL, and 25 to 500 *µ*g/mL, respectively. The regression coefficients were 0.9998, 0.9999, and 0.9991, respectively.

## 1. Introduction

As is well known, capillary electrophoresis (CE) is powerful in separation and determination as a recently emerging liquid separation technology. Because of its high efficiency, high speed, ease of automation, ease of cleaning up contaminants, and analysis of sample and reagent in small volume, CE has been used to separate many different analytes, from small ions to macromolecules such as proteins and nucleic acids, and it is even used for the separation of particles and intact cells. In recent years CE usually is widely used in content determination and quality control of the traditional Chinese medicine herbs. However, the capillary tube has narrow internal diameter, which means that the CE technique generally has a low sample capacity and a short optical path length for online UV detection. All the above could decrease the detection sensitivity. Therefore, it is necessary to develop a suitable sample concentration step prior to separation to improve the limit of detection in CE.

There are two approaches to improve the detection sensitivity of CE, the off-column and online preconcentration method [1, 2]. However, the off-line extraction introduces more steps before the CE procedure, increasing the risk of analyte losses. Most online concentration techniques are based on the velocity change in analyte between the sample zone and the separation zone and are easy to be operated. Therefore, online preconcentration, or stacking [3–6], is commonly used to improve the CE sensitivity, which increases the injected amount of analytes by using hydrodynamic or electrokinetic methods. Nowadays several methods of sample stacking have been used widely [7–10]. The methods include (i) normal stacking mode (NSM), (ii) field-amplified sample stacking (FASS), (iii) pH-mediated stacking, (iv) acetonitrile stacking, and (v) large volume sample stacking (LVSS). Among all above, LVSS is a basic and widely adopted approach to enrichment [11–15], for it is simple and feasible and is suitable for detecting low levels of materials in drugs and the limit of detection can be achieved ng/mL. And the results of LVSS analysis are in high accuracy and good precision. It needs less sample consumption and has wide range of application. In this paper, an online concentration of LVSS in capillary electrophoresis is used to improve the detection sensitivity of the analysis.

**Figure 1:**
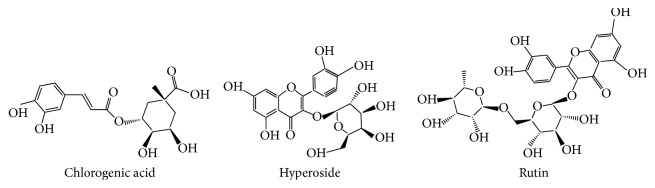
The structures of rutin, hyperoside, and chlorogenic acid.

Flos Farfarae is the flower buds of* Tussilago farfara *L., which is mostly produced in Shanxi, Henan, Gansu, and Hebei provinces and so on. As a widely used traditional Chinese medicine (TCM), Flos Farfarae has the therapeutic functions of relieving cough and reducing sputum. A variety of active compounds (triterpenoid saponins, sesquiterpenes, flavonoids, alkaloids, phthalic acid esters, chlorogenic acid, volatile oil, etc.) have been found in Flos Farfarae [16–19]. Rutin, hyperoside, and chlorogenic acid are the main active components in it and the structure of them is shown in Figure 1. In order to control the quality of Flos Farfarae better, establishing a method of the determination of rutin, hyperoside, and chlorogenic acid in Flos Farfarae is necessary. In many reports, high performance liquid chromatography (HPLC) is a good technique which has been used in the determination of compounds in Flos Farfarae [20–22]. Nowadays, Capillary electrophoresis gradually becomes a new technology in quality control of Flos Farfarae and other TCM [23–29].

In this study a new method combining the LVSS technique and high-efficiency CE was developed in order to analyze and determine three compounds in Flos Farfarae [30]. This method could relatively highly enhance the detection sensitivity of the compounds in Flos Farfarae and thus plays an important role in its quality control.

## 2. Materials and Methods

### 2.1. Reagents and Materials

Rutin, hyperoside, and chlorogenic acid were supplied by The National Institute For The Control of Pharmaceutical and Biological Products. Flos Farfarae were purchased from the Institutes for the drug Control of Taiyuan in Shanxi (S1), Linxian in Shanxi (S2), Anguo in Hebei (S3), Anyang in Anhui (S4), and Zhenzhou in Zhengzhou (S5). Borax and methanol were of analytical reagent grade. The stock solution containing rutin 5.0 mg/mL was prepared by dissolving 5.0 mg rutin with 1 mL methanol and stored in a refrigerator (4°C). The stock solution containing chlorogenic acid 4.0 mg/mL was prepared by dissolving 4.0 mg chlorogenic acid with 1 mL methanol. The stock solution containing hyperoside 3.3 mg/mL was prepared by dissolving 3.3 mg hyperoside with 1 mL methanol. The desired concentration was prepared by diluting the stock solution with methanol prior to use. All solutions were filtered through a membrane (0.45 *µ*m) and then degassed (ultrasonic) for 2 min prior to use. The pH of buffers was adjusted with sodium hydroxide.

### 2.2. Apparatus and Operating Conditions for CE

The experiments were performed on a P/ACE MDQ system (AB Sciex, CA, USA) equipped with P/ACE diode-array detector. The system was controlled by P/ACE station software. The separation was carried out in a 75 *μ*m × 50.2 cm fused-silica capillary (10.2 cm to the detector) (Yongnian Optical Fiber Factory, Hebei Province, China) with a cartridge of an 800 × 100 *μ*m detector window.

The capillary was conditioned well by being flushed with 0.1 mol/L sodium hydroxide, followed by water and methanol for 10 min at the pressure of 20 psi, respectively. Separation was carried out in reverse mode of electrode polarity, and applied voltage was maintained at 20 kV. The temperature of the capillary was maintained at 25°C and 364 nm was selected as the detection wavelength.

### 2.3. Sample Preparation

First, the sample was crushed into powder by using a Chinese herbal medicine crusher. The powder of samples (about 1.0 g) was weighed and extracted with 30 mL methanol in an ultrasonic bath for 30 min. Then the extracts were filtrated by filter paper and 0.45 *μ*m membrane filter, respectively, and analyzed under the optimized condition by CE.

### 2.4. Running Buffer for CE

The running buffer was prepared as follows: 5 mL, 80 mM NaH_2_P0_4_ was mixed with 5 mL, 80 mM borax in a 10 mL flask. And methanol of 3 mL was added to the above buffer. Thus the running buffer of 40 mM NaH_2_P0_4_-40 mM Borax-30% v/v methanol was obtained.

### 2.5. Operating Conditions for Large-Volume Sample Stacking

There are three main steps in the LVSS method [31–33]: first, the capillary was filled with the running buffer; then the standard or sample was injected by hydrodynamic injection at the pressure of 0.5 psi in short time (about 4 seconds); second, when the sample solution flows into the capillary after a certain length, reversing polarity voltage (about 25 kV) was applied; third, the sample was stacked at the interface between the buffer and the sample zone. When the current reached 95% of the original value, the applied voltage was switched to normal polarity, and the separation was finished.

## 3. Results and Discussions

### 3.1. Effect of Buffer Concentration and pH

In selecting the proper electrophoretic medium for CE, several characteristics of the solvents should be considered including viscosity, dielectric constant, electrical and thermal conductivity, self-dissociation constant, polarity, and boiling point. The commonly used running buffers included borate, phosphate, or acetate. Borate buffers usually gave better resolution of the analytes than the other buffers.

Through being investigated by phosphate, acetic acid, and borax buffer system, the mixture of borax and NaH_2_PO_4_ was discovered as the best buffer system, after peak shape and analysis time were considered comprehensively. Additionally, buffer concentration obviously affects the separation because it can influence the electroosmotic flow (EOF) and the viscosity of electrolyte. In order to obtain the best resolution, the concentration of buffer was investigated in the range from 20 to 50 mM. The resolution of the analytes increased with the buffer concentration increasing. This phenomenon may be attributed to the decreased EOF with increased ion strength (decreasing of zeta-potential). Considering the resolution and the running current, the buffer containing 40 mM borax and 40 mM NaH_2_PO_4_ was selected for the subsequent experiments.

The pH of buffer is also a governing factor in electrophoretic separation, because it determines the extent of ionization of each individual analyte and influences the properties of the inner surface of capillary. Separation systems show completely different selectivities when the pH values are different. The buffer solutions in this work were prepared by adjusting their pH value with sodium hydroxide. Considering the analysis time and resolution of the main peaks, pH 9.0 was finally selected.

### 3.2. Effect of Organic Modifier

In some cases, organic modifier, such as methanol and acetonitrile, was found to effectively improve the separation selectivity, efficiency, and resolution. The use of organic modifier could improve resolution satisfactorily. In order to separate compounds, methanol should be introduced. The effect of the percentage of methanol on resolution was also investigated by adding methanol ranging from 15% to 40% v/v into the buffer. The maximum resolution between the three compounds was obtained when the percentage of methanol in the buffer was 30% v/v.

In all, the pH, nature,and concentration of buffer and nature and concentration of organic modifier were the main factors that influence the separation of CE. So the separation was attempted in the mixture of 40 mM borax-40 mM NaH_2_PO_4_ containing 30% v/v methanol (pH 9.0) and we found that the results were satisfactory.

### 3.3. Effect of Applied Voltage

The applied voltage also can influence the migration times of analytes in CE. With increased voltage, the analysis time became shorter. In this paper, the effect of applied voltage was tested in the range of 10–30 kV. The results showed that the migration time of the compounds investigated was shortened with increased applied voltage. However, permanently physical damage of the capillary usually was caused by a high voltage in LVSS [34, 35]; thus the following CE process was blocked. This is because, during the stacking process, Joule heating was generated mainly in the sample zone. As the sample zone was driven to move back to remove solvent by the EOF, the sample zone became narrower and narrower, which brought about Joule heating being focused on a small area close to the capillary inlet in the sample zone. Applying a high voltage would produce a high electric current; thus great Joule heating was generated in this narrow area and the capillary could be destroyed itself. In our experiment, when voltage reached 25 kV, the capillary was easy to be damaged. On account of the above, and through repeated experiments, 20 kV was proper voltage to be applied for both sample stacking and next separation.

### 3.4. Effect of Injection Volume and Stacking Time

Analytes were injected into the capillary in different injection time ranging from 1 to 10 s. During the injection step, when the injection time was longer than 4 s, the sample zone would surpass the capillary window and even be pumped out of the outlet of the capillary. When the sample zone moved backwards in the stacking process, they were detected. This way, in the present LVSS method, the maximum available injection volume was the capillary volume between the inlet and the detection window [36, 37]. In our following experiment, an injection time of 4 s was used.

In order to improve the detection limits of the analytes, sample volume should be held as small as possible after stacking. And no extra dispersion should be produced. The optimum time of compounds stacking and sample matrix removal from the capillary should also be tested. In this study, the sample solution was made by hydrodynamic injection of up to 4 s at 0.5 psi; then 20 kV voltage was used for stacking and the matrix removal time of 0.7 min (the current was 95% of its maximum value) was selected.

### 3.5. Linearity, Reproducibility, and LOD

Under the optimized conditions, a series of the standard solutions were tested to determine the linearity in this method. The linear regression equations and correlation coefficients are shown in Table 1. The reproducibility of the analytes in the experiment was determined under the optimum conditions by repeated injecting standard solutions (80 *μ*g/mL, *n* = 6). The relative standard deviations (RSD) of rutin, hyperoside, and chlorogenic acid were 2.5%, 2.9%, and 2.0%, respectively. The result indicated that precision of the instrument was good. The sample solution was injected discontinuously six times within 24 h. The RSD values of peak area were 1.1%, 2.1%, and 1.7%, which indicated that the sample was stable in 24 h at room temperature. The limit of detection of rutin, hyperoside, and chlorogenic acid is 4 *μ*g/mL, 5 *μ*g/mL, and 3 *μ*g/mL, respectively, before enrichment and 0.91 *μ*g/mL, 0.94 *μ*g/mL, and 0.48 *μ*g/mL, respectively, after enrichment.

### 3.6. Determination and Accuracy of Three Compounds in Flos Farfarae

**Figure 2:**
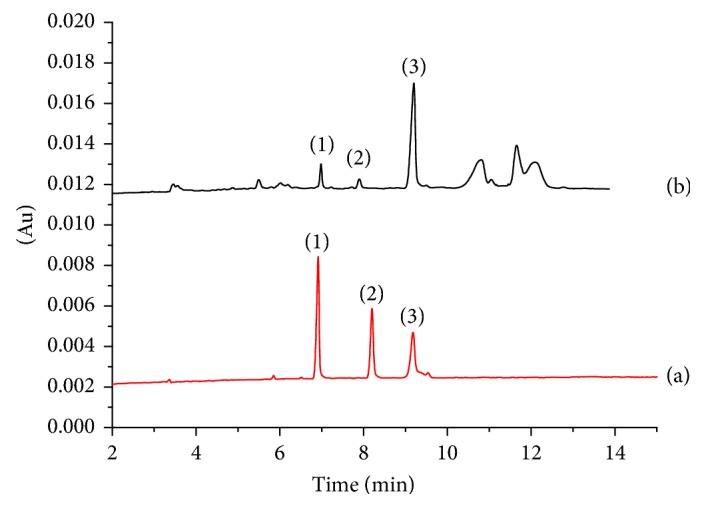
Electropherograms of (a) standard solution and (b) sample solution by CZE: (1) rutin; (2) hyperoside; (3) chlorogenic acid. CZE conditions: 40 mM NaH_2_P0_4_-40 mM borax (pH 9.0)-30% v/v methanol; voltage: 20 kV; injection volume: 0.5 psi, 4 s; detection wavelength: 364 nm; the concentration of individual compounds in standard sample: 200 ug/mL.

**Figure 3:**
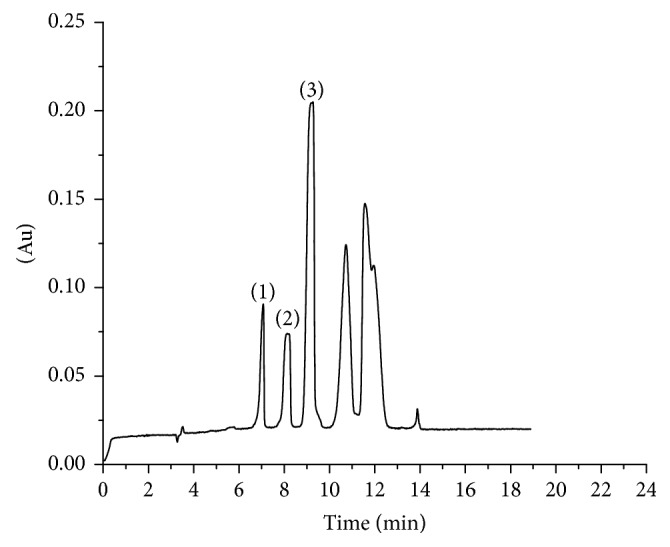
Electropherograms of sample solution by LVSS method: (1) rutin; (2) hyperoside; (3) chlorogenic acid. CE conditions: 40 mM NaH_2_P0_4_-40 mM Borax-30% v/v methanol (pH 9.0); operating voltage: 20 kV (+)-(−), the sample hydrodynamic injection of 4 s at 0.5 psi, water injection time: 40 s; detection wavelength: 364 nm.

According to the procedures described in the experimental section, a method of the LVSS combining CE has been developed for the analysis of the three compounds in Flos Farfarae samples obtained from different regions. Electropherograms of standard solution and sample solution by CZE are shown in Figure 2. Electropherogram of sample solution by LVSS method is shown in Figure 3. There was 14-, 26-, and 5-fold enrichment of rutin, hyperoside, and chlorogenic acid according to ratio of peak areas of two methods. Peak identification was performed by a standard-addition method. Standard curve method was used to calculate the contents of samples. The assay results of five samples are summarized in Table 2. The contents of three compounds in Flos Farfarae are well in agreement with several previous reports [38, 39]. Accurate amounts of standards were added to the powder drug of known content and then prepared to obtain sample solutions. Subsequently, the spiked sample solution was analyzed under the optimum conditions. The results are given in Table 3. The results showed that the proposed method is reliable, accurate, and reproducible for the determination of three compounds in Flos Farfarae.

## 4. Conclusion

In conclusion, this study demonstrates that the use of CE combining a simple large-volume sample stacking method is effective in detecting three compounds in Flos Farfarae. Successful separation and accurate results were obtained. The compound was determined with high efficiency in a short period of time (less than ten minutes). The proposed method was validated and showed a good performance with respect to selectivity, precision, linearity, and accuracy. In all, compared with the previous methods by HPLC, HPCE showed advantages as follows: analysis time shortened greatly, sensitivity and limit of detection improved for simple and practical online enrichment, pretreatment is relatively simple, and there is less sample consumption.

Therefore, this new method is promising for the quality control of Flos Farfarae in the future.

## Figures and Tables

**Table 1:** Calibration range, regression equation, and correlation coefficients.

Analytes	Calibration range (*μ*g/mL)	Regression equation	Regression coefficients
Rutin	20.0–400.0	*Y* = 408814*X* + 1475940	0.9999
Chlorogenic acid	25.0–500.0	*Y* = 161158*X* + 254880	0.9991
Hyperoside	16.5–330.0	*Y* = 207829*X* − 80269	0.9999

**Table 2:** Determination results of three compounds in Flos Farfarae (*n* = 3).

Source of sample	Content of rutin (mg/g)	Content of chlorogenic acid (mg/g)	Content of hyperoside (mg/g)
S1	5.1	9.2	2.3
S2	2.4	7.9	2.1
S3	3.1	10.2	3.3
S4	5.8	11.1	3.4
S5	4.7	10.0	2.4

**Table 3:** Recovery of rutin, chlorogenic acid, and hyperoside in Flos Farfarae.

Samples	Original amount (mg)	Added amount (mg)	Found amount (mg)	Average recovery (%)	RSD (%)
Rutin	1.273	1.300	2.578	100.3	0.1
Chlorogenic acid	2.114	2.100	4.230	100.0	3.5
Hyperoside	2.323	2.300	4.636	100.5	1.9
